# Risk of preeclampsia by gestational weight gain in women with varied prepregnancy BMI: A retrospective cohort study

**DOI:** 10.3389/fendo.2022.967102

**Published:** 2022-10-14

**Authors:** Xiaoli Gong, Jiaxin Li, Yuanhui Jiang, Pengbo Yuan, Lian Chen, Yike Yang, You Li, Mengxing Sun, Yangyu Zhao, Huifeng Shi, Yuan Wei

**Affiliations:** ^1^ Department of Obstetrics and Gynecology, Peking University Third Hospital, Beijing, China; ^2^ National Clinical Research Centre for Obstetrics and Gynecology, Beijing, China; ^3^ National Centre for Healthcare Quality Management in Obstetrics, Beijing, China

**Keywords:** pregnancy, preeclampsia, body mass index, gestational weight gain, cohort study

## Abstract

**Introduction:**

Despite the important clinical significance, limited data on the joint contribution of prepregnancy body mass index (BMI) and gestational weight gain (GWG) to preeclampsia, the second leading cause of maternal mortality worldwide. This study aimed to estimate the risk of preeclampsia by GWG among women with varied prepregnancy BMI.

**Methods:**

We conducted a retrospective cohort study using data of 117 738 singleton pregnant women aged 18–49 years from 150 maternity hospitals in China between 2015 and 2018. GWG was calculated as the measured weight at the time of preeclampsia assessment minus prepregnancy weight; GWG velocity was calculated as the GWG divided by the gestational age at weighing. The non-linear associations of GWG with preeclampsia were examined by restricted cubic spline regression analysis according to prepregnancy BMI. The association of the GWG categories with preeclampsia was further examined by performing robust Poisson regression stratified by the prepregnancy BMI categories.

**Results:**

Among participants, 2426 (2.06%) were diagnosed with preeclampsia. Compared to women with normal BMI, those who were overweight and obese had 1.92- fold (95%CI, 1.73–2.14) and 5.06- fold (95%CI, 4.43–5.78) increased risks for preeclampsia, respectively. The association of GWG velocity with preeclampsia was presented as a J-shaped curve with the varied inflexion point (where the rate of preeclampsia was 2%), which was 0.54, 0.38, and 0.25 kg/week in women with normal BMI, overweight, and obesity, respectively; a steep risk rise was observed along with GWG velocity beyond the inflexion points. The overall adjusted relative risk for preeclampsia was calculated among women with the different GWG categories of GWG.

**Conclusions:**

The findings highlight that high prepregnancy BMI and exceed GWG contributed to increased risk of preeclampsia with a superimposed effect and underscore the need to optimize the recommendations for GWG for women with different prepregnancy BMI.

## Introduction

Preeclampsia, commonly defined as new-onset hypertension and proteinuria after 20 weeks of gestation (1), is the second leading cause of maternal mortality and a major contributor of maternal and perinatal morbidity worldwide (2). Preeclampsia complicates 2–8% of pregnancies (1, 3). Given no known cure for preeclampsia other than delivery, identifying the risk factors is critical for developing prevention strategies to reduce the incidence of preeclampsia and consequent adverse outcomes.

Overweight and obesity, the well documented risk factor for preeclampsia (4–10), has seen a rising trend since 1980 all over the world (11, 12). The World Health Organization (WHO) estimates that the global age-standardized mean body mass index (BMI) increased from 22 kg/m^2^ in 1975 to 24.6 kg/m^2^ in 2016 in adult women, with the global age-standardized prevalence of overweight and obesity increased from 22.7% and 6.3% to 39.2% and 15.1% in adult women, respectively (12). In China, the annual increase in age-standardized mean BMI increased 0.09 kg/m² for adult women in 14 years from 2004, leading to virtually identical age-standardized mean BMIs of 24.1 kg/m², overweight prevalence of 36.7% and obesity prevalence of 7.2% in 2018 (13). The rise in mean BMI was faster in women aged 18-29, the main fertility population (13).

Weight gain during pregnancy may vary among pregnant women with different prepregnancy BMI and excessive gestational weight gain (GWG) is more likely to occur in women who are overweight and obese (14). However, the relationship between GWG and preeclampsia is inconclusive due to limited evidence (15, 16). In clinical practice, it is more valuable to estimate the joint contribution of prepregnancy BMI and GWG to the occurrence of preeclampsia (17), but this has rarely been investigated (15, 18). This study therefore aimed to estimate the risk of preeclampsia by gestational weight gain among women with varied prepregnancy BMI, by using data from a retrospective, multicenter cohort data.

## Methods

### Study design and participants

A prospective cohort study determining the factors of preeclampsia had been conducted at 180 hospitals across 23 provinces in China between 2015 and 2018. Pregnant women who had been registered for antenatal care in the hospitals were recruited to participate. Our study population was drawn from singleton pregnant women aged 18–49 years with preeclampsia assessment at 20–40 weeks in the cohort database. We excluded women with prepregnancy hypertension, diabetes mellitus, and those without available data of height, prepregnancy weight, and gestational weight measurement. Finally, 117 738 pregnant women with required data from 150 maternity hospitals were included (**Figure S1**).

### Prepregnancy BMI and GWG

Prepregnancy BMI was calculated as the self-reported prepregnancy weight in kilograms (kg) divided by height in meters squared measured at the first time of antenatal care. Subsequently, prepregnancy BMI was then classified as underweight [BMI: <18.5 kg/m^2^], normal weight [BMI: 18.5–23.9 kg/m^2^], overweight [BMI: 24–27.9 kg/m^2^], obesity [BMI: ≥ 28 kg/m^2^] by using the diagnostic criteria in Chinese adults (19).

Pregnant women were weighed at routine antenatal care visits. GWG was calculated as the measured weight at the time of preeclampsia assessment, which was represented by the measured weight at the last assessment time if all preeclampsia assessments (≥1 times) were negative or at the time of being firstly diagnosed with preeclampsia, minus prepregnancy weight. We calculated GWG velocity as the GWG divided by gestational age at weighing, in order to account for that greater weight gain may be observed at later measurement. Given no previously-published data of BMI-specific weight-gain-for-gestational-age for Chinese population, this is a reasonable approach because previous studies have shown that weight gain during pregnancy is roughly linear (20).

### Diagnosis on preeclampsia

Preeclampsia assessment had been conducted by obstetricians in the original prospective cohort by using the criteria recommended by the Chinese Society of Obstetrics and Gynecology (21). Preeclampsia cases were identified if a woman had new onset hypertension at or after 20 weeks of gestation, accompanied by one of the following: proteinuria, other maternal organ dysfunction (including heart, lung, liver, kidney), or hematological, digestive, and neurological involvement, and/or uteroplacental dysfunction (21).

### Covariates

Covariates including year, geographical region, age, ethnic origin, education, Hukou (urban residents, rural residents, or rural-to-urban migrants), mode of conception, and primigravida were drawn from medical records.

### Statistical analysis

Age was described as mean and standard deviation (SD) and comparisons between women with preeclampsia and those without preeclampsia were performed using the Student’s t test. Categorical variables, including year, region, ethnic origin, education, hukou, mode of conception, and primigravida, were described as counts with percentages and comparisons in the two groups were performed using Chi-squared test.

GWG (<6.5, 6.5–9.9, 10.0–13.4, 13.5–16.9, 17.0–19.9, and ≥ 20.0 kg) and GWG velocity (<0.23, 0.23–0.32, 0.33–0.42, 0.43–0.52, 0.53–0.59, ≥0.60 kg/week) were divided into six groups according to the 25^th^, 50^th^, 75^th^, 90^th^, and 95^th^ percentiles. The incidence of preeclampsia was calculated within each combination of prepregnancy BMI categories and GWG or GWG velocity categories.

The non-linear associations of GWG and GWG velocity with preeclampsia were examined by employing logistic regression models with restricted cubic splines. GWG and GWG velocity were modelled using restricted cubic splines; knots were determined according to the principle of minimized Akaike Information Criterion (AIC) (22). Sensitivity analyses were performed by adjusting for different covariates. Model 1 adjusted for no covariate. Model 2 adjusted for the covariates. Model 3 additionally adjusted for prepregnancy BMI. Similar methods were used to determine the association of prepregnancy BMI with preeclampsia and GWG velocity were additionally adjusted for in model 3. We also determined the association of GWG and GWG velocity with preeclampsia given prepregnancy BMI categories (underweight, normal weight, overweight, and obesity) by employing logistic regression models with restricted cubic splines which adjusted for covariates as model 2. Predicted absolute probabilities of preeclampsia with 95% confidence intervals (CIs) were calculated with respect to prepregnancy BMI, GWG and GWG velocity based on these models.

The associations were further examined by performing multivariable robust Poisson regression models, in which the categorical variables of GWG/GWG velocity and prepregnancy BMI were used. We also performed a stratified analysis by the prepregnancy BMI categories. All models adjusted for the covariates. Relative risks (RRs) and the 95% CIs were calculated by these models.

All statistical analyses were performed with SAS software, version 9.0 and the R statistical software, version 3.6.2. A two-tailed P-value < 0.05 was considered statistically significant.

## Results

**Figure 1:**
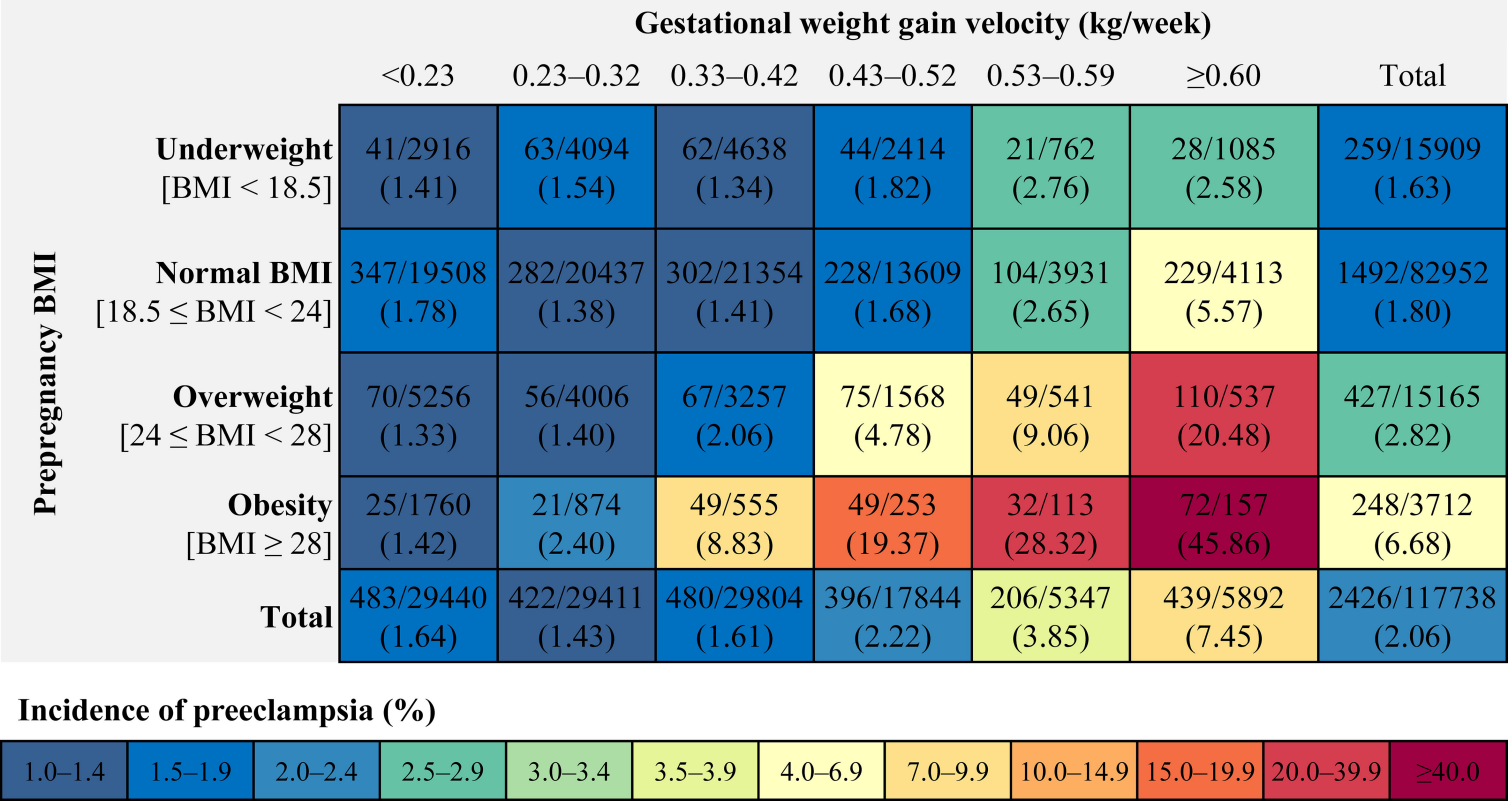
Incidence of preeclampsia within each combination of prepregnancy BMI categories and categories of gestational weight gain velocity. BMI, body mass index.

Among 117 738 pregnant women (mean [SD] age, 28.9 [4.4] years) included in the study, 2426 (2.06%) were diagnosed with preeclampsia (**Table 1**). The incidence of preeclampsia was 1.63% in women with underweight, 1.80% in women with normal weight, 2.82% in women with overweight, and 6.68% in women with obesity. The incidence of preeclampsia was 1.64%, 1.43%, 1.61%, 2.22%, 3.85% and 7.45% in women with pregnancy weight gain of < 0.23, 0.23 – 0.32, 0.33 – 0.42, 0.43 – 0.52, 0.53 – 0.59 and ≥ 0.60 kg/week. The incidence ranged from 1.33% to 45.86% for preeclampsia in all combinations of prepregnancy BMI categories and GWG velocity categories; it increased across the full range of GWG velocity categories from the group of < 0.23 kg/week to the group of ≥ 0.60 kg/week (**Figure 1**). Similar association were found in combinations of prepregnancy BMI categories and GWG categories (**Figure S2**).

**Table 1:** Basic characteristics of participants.

	Non-preeclampsia	Preeclampsia	Total
	(n = 115312)	(n = 2426)	(n = 117738)
Year, No. (%)			
2015	24911 (21.6)	367 (15.1) *	25278 (21.5)
2016	44560 (38.6)	1097 (45.2)	45657 (38.8)
2017	33516 (29.1)	781 (32.2)	34297 (29.1)
2018	12325 (10.7)	181 (7.5)	12506 (10.6)
Region, No. (%)			
Eastern China	59698 (51.8)	1075 (44.3) *	60773 (51.6)
Central China	16515 (14.3)	623 (25.7)	17138 (14.6)
Western China	39099 (33.9)	728 (30.0)	39827 (33.8)
Age, Mean (SD)	28.9 (4.3)	29.1 (4.6) *	28.9 (4.4)
Ethnic origin, No. (%)			
Han	113090 (98.1)	2386 (98.4)	115476 (98.1)
Other	2222 (1.9)	40 (1.6)	2262 (1.9)
Education, No. (%)			
High school	42716 (37.0)	1073 (44.2) *	43789 (37.2)
College	33995 (29.5)	801 (33.0)	34796 (29.6)
Master	6924 (6.0)	102 (4.2)	7026 (6.0)
Other	31677 (27.5)	450 (18.5)	32127 (27.3)
Primigravida, No. (%)	59127 (51.3)	1264 (52.1)	60391 (51.3)
Assisted reproductive technology, No. (%)	1040 (0.9)	41 (1.7) *	1081 (0.9)
Hukou, No. (%)			
Urban residents	90838 (78.8)	1982 (81.7)	92820 (78.8)
Migrants	6924 (6.0)	161 (6.6)	7085 (6.0)
Rural residents	17550 (15.2)	283 (11.7)	17833 (15.1)
Prepregnancy body mass index (BMI), No. (%)			
Underweight [BMI < 18.5]	15650 (13.6)	259 (10.7) *	15909 (13.5)
Normal BMI [18.5 ≤ BMI < 24]	81460 (70.6)	1492 (61.5)	82952 (70.5)
Overweight [24 ≤ BMI < 28]	14738 (12.8)	427 (17.6)	15165 (12.9)
Obesity [BMI ≥ 28]	3464 (3.0)	248 (10.2)	3712 (3.2)

*P value was less than 0.05 when the characteristics were compared between women with preeclampsia and those without preeclampsia.Bold values of RRs with 95%CIs and P-values were statistically significant.

**Figure 2:**
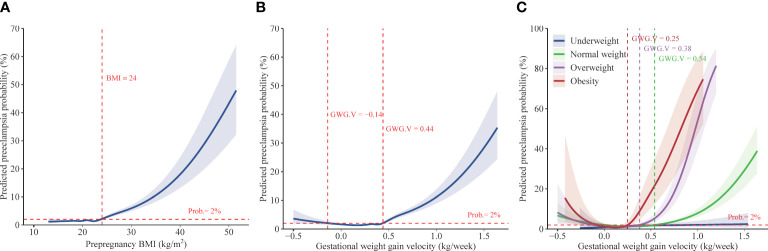
Predicted preeclampsia probabilities with respect to prepregnancy BMI and gestational weight gain velocity. Data are from China, 2015 to 2018. Predicted preeclampsia probabilities with 95% CIs were calculated with respect to prepregnancy BMI **(A)** and gestational weight gain velocity **(B)** by performing logistic regression models with restricted cubic splines. Models adjusted for year, age, education, ethnic origin, region, Hukou, assisted reproductive technology, primigravida, and prepregnancy BMI (in **Figure 2B**)/gestational weight gain velocity (in **Figure 2A**). Predicted preeclampsia probabilities with 95% CIs were also calculated **(C)** with respect to gestational weight gain velocity given varied prepregnancy BMI (underweight, normal weight, overweight, and obesity), by using performing logistic regression models with restricted cubic splines which adjusted for year, age, education, ethnic origin, region, Hukou, assisted reproductive technology, and primigravida. BMI, body mass index. GWG.V, gestational weight gain velocity. Prob., predicted preeclampsia probability.

Using restricted cubic spline regression analysis, we found that the association of prepregnancy BMI with the risk of preeclampsia is presented as an inverse L-shaped curve with an inflexion point at 24 kg/m^2^; higher prepregnancy BMI was associated with increased risk of preeclampsia beyond the inflexion point (**Figure 2A**). Adjusting for different covariates did not substantially influence the estimates (**Figure S3A**). The results of multivariable adjusted robust Poisson regression show that compared with women with normal weight, those who were overweight and obese had 1.92- fold (95%CI, 1.73–2.14) and 5.06- fold (95%CI, 4.43–5.78) increased risks for preeclampsia, respectively (**Table 2**). There was no significant difference in the risk of preeclampsia between pregnant women with underweight and those with normal weight. Sensitivity analyses adjusting for different covariates indicate the robustness of estimates (**Table 2**).

**Table 2:** Adjusted relative risks (95% CI) for preeclampsia according to prepregnancy BMI.

	**Model A**		**Model B**	
	aRR (95%CI)	p value	aRR (95%CI)	p value
**Prepregnancy body mass index (BMI)**				
Underweight [BMI < 18.5]	0.99 (0.87, 1.13)	0.876	0.92 (0.81, 1.06)	0.246
Normal BMI [18.5 ≤ BMI < 24]	1.00 [Reference]		1.00 [Reference]	
Overweight [24 ≤ BMI < 28]	**1.75 (1.57, 1.94)**	**<0.001**	**1.92 (1.73, 2.14)**	**<0.001**
Obesity [BMI ≥ 28]	**4.54 (3.98, 5.18)**	**<0.001**	**5.06 (4.43, 5.78)**	**<0.001**

CI, confidence interval. Data are from China, 2015 to 2018. Adjusted relative risks (95% CI) for preeclampsia were calculated with respect to prepregnancy BMI groups by performing robust Poisson regression models. Model A adjusted for basic characteristics of women, including year, region, age, education, ethnic origin, Hukou, assisted reproductive technology, and primigravida,. Model B additionally adjusted for gestational weight gain velocity.Bold values of RRs with 95%CIs and P-values were statistically significant.

The associations between GWG velocity and the risk of preeclampsia present a J-shaped curve with the lowest risk of less than 2% at GWG velocity of -0.14–0.44 kg/week and a sharply increased risk with GWG velocity beyond the GWG velocity range, according to restricted cubic spline regression analysis (**Figure 2B**). Adjusting for different covariates did not substantially influence the estimates (**Figure S3B**). Multivariable robust Poisson regression show that higher pregnancy weight gain velocity was associated with increased risks of preeclampsia. The adjusted relative risk was 1.20 (95%CI, 1.05–1.36), 1.55 (95%CI, 1.35–1.79), 2.73 (95%CI, 2.32–3.22), and 4.87 (95%CI, 4.27–5.56) in women with GWG velocity of 0.33–0.42, 0.43–0.52, 0.53–0.59, and ≥ 0.60 kg/week, respectively, compared with women with GWG of 0.23–0.32 kg/week (**Table 3**).

**Table 3:** Adjusted relative risks (95% CI) for preeclampsia according to GWG velocity stratified by prepregnancy BMI.

	Underweight [BMI < 18.5]		Normal BMI [18.5 ≤ BMI < 24]		Overweight [24 ≤ BMI < 28]		Obesity [BMI ≥ 28]		Total	
	(n = 15909)		(n = 82952)		(n = 15165)		(n = 3712)		(n = 117738)	
	aRR (95%CI)^*^	p value	aRR (95%CI)^*^	p value	aRR (95%CI)^*^	p value	aRR (95%CI)^*^	p value	aRR (95%CI)^†^	p value
**GWG velocity (Kg/week)**										
<0.23	0.81 (0.55, 1.20)	0.287	**1.23 (1.06, 1.44)**	**0.008**	0.98 (0.69, 1.40)	0.932	0.58 (0.33, 1.04)	0.067	0.98 (0.86, 1.12)	0.808
0.23–0.32	1.00 [Reference]		1.00 [Reference]		1.00 [Reference]		1.00 [Reference]		1.00 [Reference]	
0.33–0.42	0.95 (0.67, 1.34)	0.764	1.05 (0.89, 1.23)	0.581	**1.43 (1.01, 2.04)**	**0.044**	**3.56 (2.17, 5.83)**	**<0.001**	**1.20 (1.05, 1.36)**	**0.007**
0.43–0.52	1.24 (0.85, 1.80)	0.257	1.13 (0.94, 1.35)	0.188	**3.08 (2.20, 4.31)**	**<0.001**	**6.58 (4.05, 10.70)**	**<0.001**	**1.55 (1.35, 1.79)**	**<0.001**
0.53–0.59	**1.65 (1.02, 2.66)**	**0.039**	**1.89 (1.51, 2.36)**	**<0.001**	**5.68 (3.92, 8.23)**	**<0.001**	**8.84 (5.39, 14.50)**	**<0.001**	**2.73 (2.32, 3.22)**	**<0.001**
≥0.60	1.13 (0.73, 1.75)	0.595	**3.57 (2.99, 4.25)**	**<0.001**	**12.05 (8.91, 16.29)**	**<0.001**	**12.99 (8.22, 20.51)**	**<0.001**	**4.87 (4.27, 5.56)**	**<0.001**

BMI, body mass index; CI, confidence interval; GWG, gestational weight gain; RR, relative risk. *Adjusted relative risks (95% CI) for preeclampsia were calculated with respect to categories of gestational weight gain velocity stratified by prepregnancy BMI groups by performing robust Poisson regression models, which adjusted for basic characteristics of women, including year, region, age, education, ethnic origin, Hukou, assisted reproductive technology, and primigravida. ^†^Additionally adjusted for prepregnancy BMI groups. Data are from China, 2015 to 2018.

According to the results of stratified analysis using restricted cubic spline regression, the association of GWG velocity with preeclampsia was also presented as a J-shaped curve with the varied inflexion point (where the risk of preeclampsia was 2%) in women who were normal weight (0.54 kg/week), overweight (0.38 kg/week), and obese (0.25 kg/week); a steep rise in the risk of preeclampsia was observed along with GWG velocity beyond the inflexion points (**Figure 2C**). Multivariable robust Poisson regression show that, after adjusting for confounders, higher GWG velocity beyond the inflexion points was associated with increased risks of preeclampsia in women who were normal weight, overweight, and obese (**Table 3**). No significant association was observed between GWG velocity and the risk of preeclampsia in women with underweight. Similar association were found when employing the variable “GWG” (**Table S1** and **Figure S3C-D**).

## Discussion

In this large cohort, we found that prepregnancy overweight and obesity was strongly associated with preeclampsia. Furthermore, the J-shaped association of GWG velocity with the risk of preeclampsia was observed in women with prepregnancy BMI of ≥ 18.5 and a sharply increased risk was observed with higher pregnancy weight gain velocity beyond the inflexion point, which varied in women who were normal weight, overweight, and obese. Our findings highlight the joint contribution of prepregnancy BMI and GWG to the occurrence of preeclampsia.

Consistent with previous findings, our study confirmed that pregnant women who were overweight and obese had a higher risk of preeclampsia while women with underweight had a lower or similar risk compared to those with normal BMI (5–7, 9, 10, 23). To date, there are few studies that have investigated the association between GWG and preeclampsia, especially given varied prepregnancy BMI (24). Our study supports evidences from previous observations (10, 15, 17, 25), and contribute to a deeper understanding of the association. We found that for women with prepregnancy BMI ≥ 18.5, the association of GWG velocity with preeclampsia was presented as J-shaped curve and the risk of preeclampsia increased along with GWG beyond a threshold. Higher prepregnancy BMI and GWG not only resulted in higher risk of preeclampsia independently, but also had superimposed effect (15, 26–30). An overweight and obese prepregnancy BMI coupled with excessive GWG resulted in a multiplicative increase in the risk of preeclampsia.

Notably, the threshold for GWG for preeclampsia morbidity was lower as prepregnancy BMI increased. In our study, the threshold of GWG, beyond which risk of preeclampsia increased, was 0.54, 0.38, and 0.25 kg/week in women who were normal weight, overweight, and obese, respectively. The thresholds are consistent with the upper limit of GWG recommended by Chinese Nutrition Society in pregnant women with corresponding prepregnancy BMI (31). As recommended in *Weight Monitoring and Evaluation during Pregnancy Period of Chinese Women* by Chinese Nutrition Society (31), the mean (range) of GWG was 0.46 (0.37–0.56), 0.37 (0.26–0.48), 0.30 (0.22–0.37), and 0.22 (0.15–0.30) kg/week in women with underweight, normal weight, overweight, and obesity during the second and third trimester of pregnancy, respectively. The findings further confirm the important influence of BMI and GWG on pregnancy outcome.

We found no significant association between GWG and the risk of preeclampsia in women with prepregnancy underweight BMI. There is little published data on this association and existing studies offer contradictory findings (10, 32, 33). A population-based cohort survey of 98,820 women with singleton pregnancies in Slovenia found that excessive GWG was associated with increased odds of preeclampsia in all pre-pregnancy BMI categories, especially in underweight women (33). However, in an individual participant data meta-analysis of 265 270 pregnancies from 39 cohorts in Europe, North America, and Oceania, the significant association between GWG and preeclampsia risk in pregnant women with underweight was not found (10). More research on this topic is needed. In addition, insufficient gestational weight gain was found to be associated with increased risk of preeclampsia among women with normal weight in our study. The result was supported by a retrospective study showed that low GWG could lead to severe maternal morbidity, including eclampsia postpartum (24). Unfortunately, we did not distinguish the types of preeclampsia and weight gain in different pregnancy period. A cohort study of 62705 nulliparous women from Sweden indicates that higher GWG was much more strongly associated with late-onset preeclampsia than early-onset preeclampsia (16).

The mechanism by which prepregnancy obesity and excessive GWG result in preeclampsia had not been fully clarified, but studies suggested that oxidative stress may play an important role in the pathogenesis. High prepregnancy BMI and excessive GWG might increase the level of oxidative stress, induce systemic inflammation and accelerate damage to vascular endothelial cells, resulting in preeclampsia (15, 34–36). Pregnant women with normal prepregnancy BMI and low GWG might be accompanied by insufficient intake of essential nutrients such as antioxidant vitamins and calcium, which would increase the risk of preeclampsia (37–41).

This finding has important implications for developing weight management strategies before and during pregnancy. Controlling excessive GWG was recommended to reduce the risk of preeclampsia for women with prepregnancy BMI of ≥ 18.5, although the idea needs confirmation by RCTs. Our finding that the association of GWG velocity with preeclampsia was presented as a J-shaped curve with the varied inflexion point in women with normal weight, overweight, and obesity underscores the need to optimize the recommendations for weight gain during pregnancy for different BMI groups to reduce the risk of preeclampsia. The recommendation is consistent with the GWG recommendation of the Chinese Nutrition Society (31). For women who are overweight and obese, reducing weight before pregnancy can help reduce the incidence of preeclampsia, which was supported by a cohort study of 436,414 women with singleton gestations (42).

Our study has several limitations. The prepregnancy weight and height was self-reported by pregnant women, which would lead to exposure misclassification, However, previous findings indicate that self-reported prepregnancy weight can be used for calculation of BMI and GWG when an early measurement of weight during pregnancy is not available (43). Additionally, because there are not pregnant weight gain z-score charts for Chinese women, we used GWG velocity to induce bias caused by the inherent correlation between weight gain and gestational duration (longer pregnancies have more opportunity to gain weight). However, it may do not eliminate the bias to an adequate extent. Finally, we failed to collect information of other risk factors of preeclampsia, such as smoking which has an estimated prevalence of 2.2% in Chinese women (44); and also failed to collect the indicators of preeclampsia, such as blood pressure.

## Conclusion

In conclusion, we found that high prepregnancy BMI and exceed GWG contributed to increased risk of preeclampsia with a superimposed effect. Prepregnancy overweight and obesity was strongly associated with preeclampsia. Furthermore, the J-shaped association of GWG velocity with the risk of preeclampsia was observed in women with prepregnancy BMI of ≥ 18.5 and a steep rise in the risk of preeclampsia was observed along with higher GWG velocity beyond the inflexion point, which was 0.54, 0.38, and 0.25 kg per week in women with normal weight, overweight, and obesity, respectively. Reducing weight before pregnancy and controlling excessive GWG are recommended for women who are overweight and obese to reduce the risk of preeclampsia. Our findings also underscore the need to optimize the recommendations for GWG for women with different prepregnancy BMI.

## Data availability statement

The original contributions presented in the study are included in the article/**Supplementary Material**. Further inquiries can be directed to the corresponding authors.

## Ethics statement

The studies involving human participants were reviewed and approved by the Peking University Third Hospital Medical Science Research Ethics Committee. The patients/participants provided their written informed consent to participate in this study.

## Author contributions

Conceptualization, HS, XG, and JL; methodology, HS, XG, and JL; validation, YJ, PY, LC, YY, YL, and MS; formal analysis, HS, XG, and JL; investigation, YJ, PY, LC, and YY; resources, YW; data curation, YL and MS; writing—original draft preparation, HS, XG, and JL; writing—review and editing, PY, LC, YY, YL, MS, YW, and YZ; supervision, YW, HS, and YZ; project administration, YW and YZ; funding acquisition, YW and YZ. All authors have read and approved the final manuscript.

## Funding

This research was funded by grants from National Key Research and Development Program (grant number 2021YFC2700700 [YW]) and Peking University Third Hospital Cohort Building Program (BYSYDL2019001 [YZ]). The funders had no role in the design of the study; in the collection, analyses, or interpretation of data; in the writing of the manuscript, or in the decision to publish the results.

## Acknowledgments

We thank all hospitals and health workers who contributed to the data collection and management, as well as the pregnant women who participated in our study.

## Conflict of interest

The authors declare that the research was conducted in the absence of any commercial or financial relationships that could be construed as a potential conflict of interest.

## Publisher’s note

All claims expressed in this article are solely those of the authors and do not necessarily represent those of their affiliated organizations, or those of the publisher, the editors and the reviewers. Any product that may be evaluated in this article, or claim that may be made by its manufacturer, is not guaranteed or endorsed by the publisher.
