# Supplementary material for: Risk of preeclampsia by gestational weight gain in women with varied prepregnancy BMI: A retrospective cohort study
Source: Front Endocrinol (Lausanne). 2022 Oct 14;13:967102. doi: 10.3389/fendo.2022.967102 (PMC9616116; doi:10.3389/fendo.2022.967102)
Supplement: Supplementary file 1 [file DataSheet_1.docx]

**Risk of Preeclampsia by Gestational Weight Gain in Women with Varied Prepregnancy BMI: A Retrospective Cohort Study**

**Supplementary file**

**
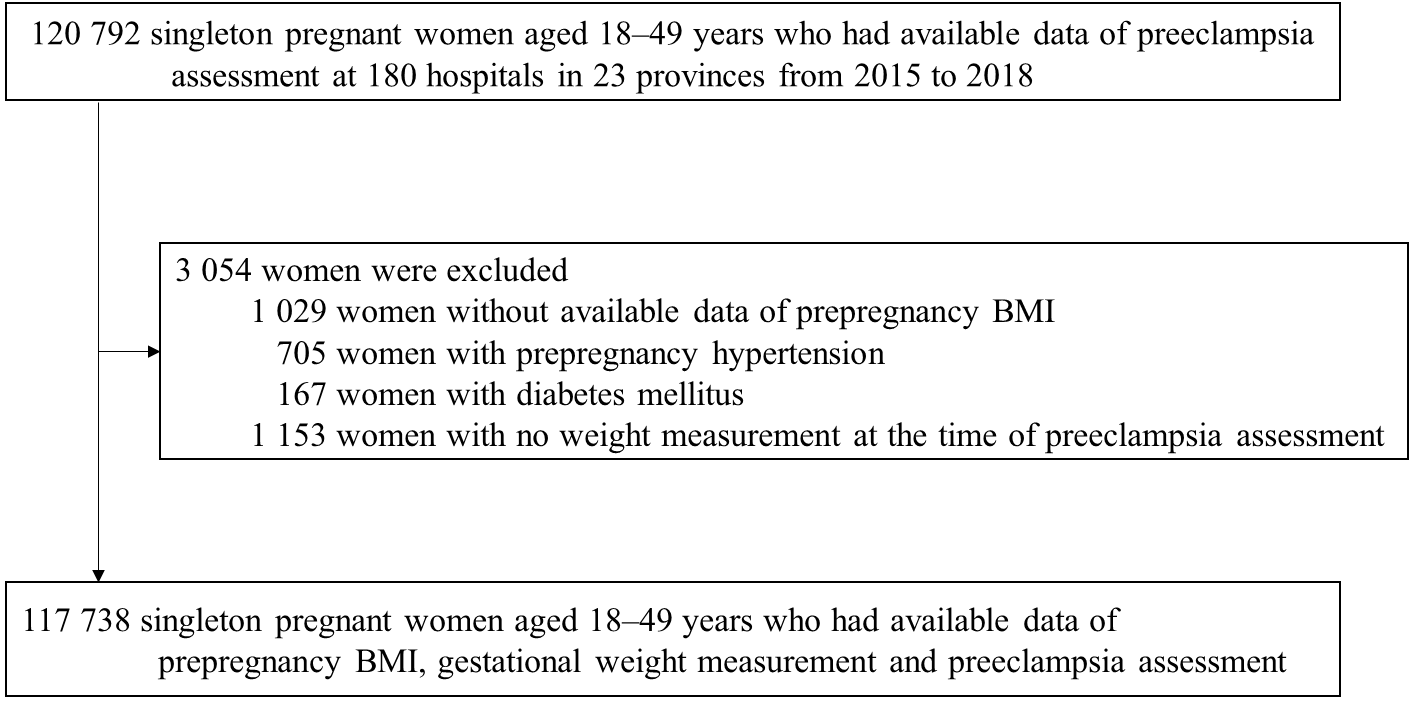
**

**Figure S1** Study profile. BMI, body mass index.

|  |  | **Gestational weight gain (Kg)** | | | | | | |
| --- | --- | --- | --- | --- | --- | --- | --- | --- |
|  |  | <6.5 | 6.5–9.9 | 10.0–13.4 | 13.5–16.9 | 17.0–19.9 | ≥20.0 | Total |
| **Prepregnancy BMI** | **Underweight**  [BMI < 18.5] | 55/3245 (1.69) | 73/4420 (1.65) | 60/3668 (1.64) | 41/2795 (1.47) | 17/952 (1.79) | 13/829 (1.57) | 259/15909 (1.63) |
|  | **Normal BMI**  [18.5 ≤ BMI < 24] | 374/20061 (1.86) | 322/24111 (1.34) | 283/17683 (1.60) | 231/13311 (1.74) | 109/4600 (2.37) | 173/3186 (5.43) | 1492/82952 (1.80) |
|  | **Overweight**  [24 ≤ BMI < 28] | 60/4942 (1.21) | 83/4390 (1.89) | 77/2840 (2.71) | 64/1847 (3.47) | 43/658 (6.53) | 100/488 (20.49) | 427/15165 (2.82) |
|  | **Obesity**  [BMI ≥ 28] | 24/1639 (1.46) | 43/991 (4.34) | 61/551 (11.07) | 33/292 (11.30) | 20/100 (20.00) | 67/139 (48.20) | 248/3712 (6.68) |
|  | **Total** | 513/29887 (1.72) | 521/33912 (1.54) | 481/24742 (1.94) | 369/18245 (2.02) | 189/6310 (3.00) | 353/4642 (7.60) | 2426/117738 (2.06) |

**Incidence of preeclampsia (%)**

| 1.0–1.4 | 1.5–1.9 | 2.0–2.4 | 2.5–2.9 | 3.0–3.4 | 3.5–3.9 | 4.0–6.9 | 7.0–9.9 | 10.0–14.9 | 15.0–19.9 | 20.0–39.9 | ≥40.0 |
| --- | --- | --- | --- | --- | --- | --- | --- | --- | --- | --- | --- |

**Figure S2** Incidence of preeclampsia within each combination of prepregnancy BMI categories and gestational weight gain categories. BMI, body mass index.


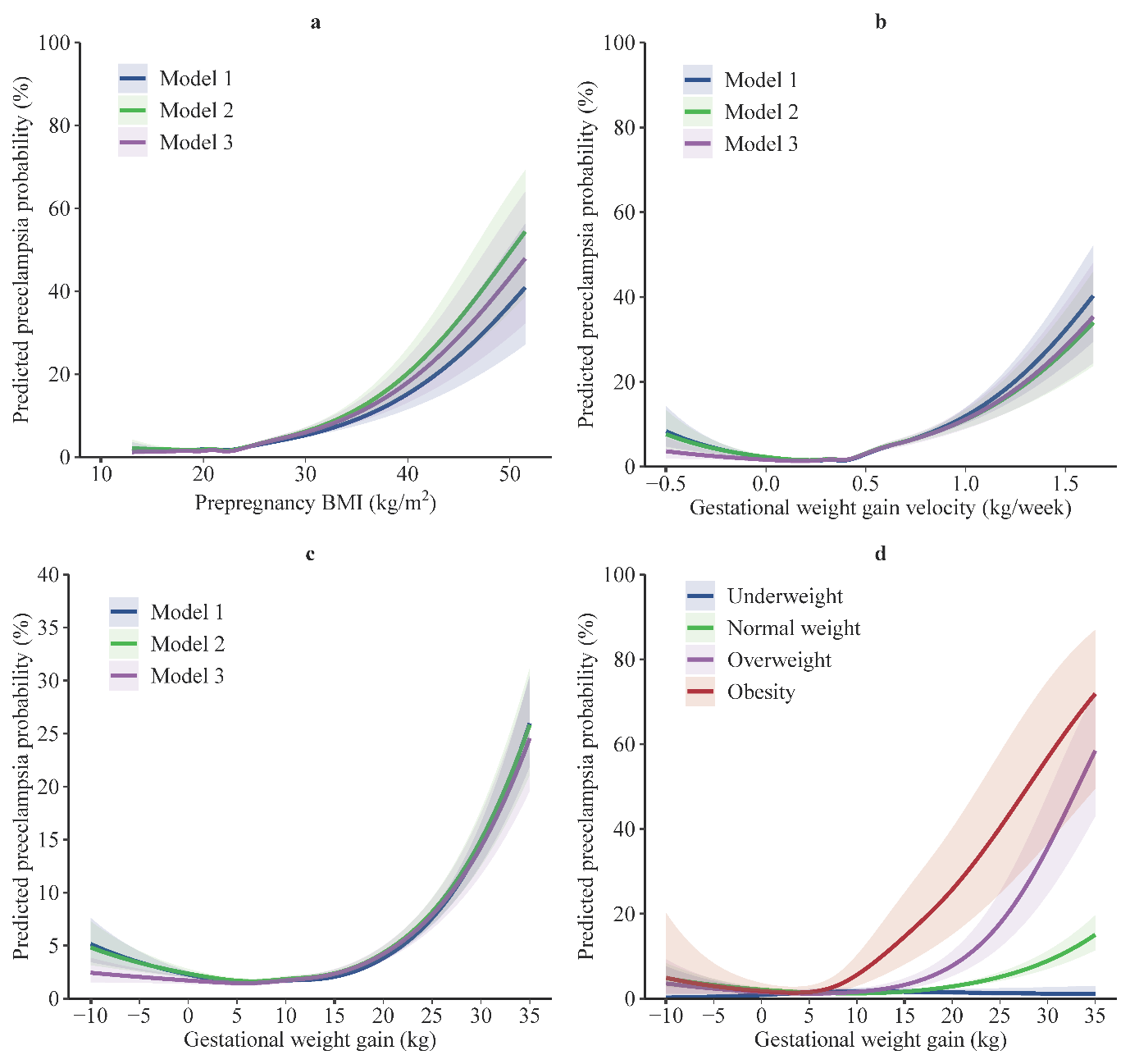


**Figure S3** Predicted preeclampsia probabilities with respect to prepregnancy BMI and gestational weight gain. Data are from China, 2015 to 2018.

Predicted preeclampsia probabilities with 95% CIs were calculated with respect to prepregnancy BMI (a), gestational weight gain velocity (b), and gestational weight gain (c) by performing logistic regression models with restricted cubic splines. Model 1 adjusted for no covariate. Model 2 adjusted for basic characteristics of women, including year, age, education, ethnic origin, region, Hukou, assisted reproductive technology, and primigravida. Model 3 additionally adjusted for gestational weight gain velocity in Figure S3a or prepregnancy BMI in Figure S3b and S3c. (d) Predicted preeclampsia probabilities with 95% CIs were calculated by using Model 3 with respect to gestational weight gain given varied prepregnancy BMI (underweight, normal weight, overweight, and obesity).

**Table** **S1** Adjusted relative risks (95% CI) for preeclampsia according to gestational weight gain stratified by prepregnancy BMI

|  | Underweight  [BMI < 18.5] | |  | Normal BMI  [18.5 ≤ BMI < 24] | |  | Overweight  [24 ≤ BMI < 28] | |  | Obesity  [BMI ≥ 28] | |  | Total | |
| --- | --- | --- | --- | --- | --- | --- | --- | --- | --- | --- | --- | --- | --- | --- |
|  | (n = 15909) | |  | (n = 82952) | |  | (n = 15165) | |  | (n = 3712) | |  | (n = 117738) | |
|  | aRR (95%CI)^*^ | p value |  | aRR (95%CIs) ^*^ | p value |  | aRR (95%CIs) ^*^ | p value |  | aRR (95%CIs) ^*^ | p value |  | aRR (95%CIs) ^†^ | p value |
| **Gestational weight gain (kg)** | | | | | | | | | | | | | | |
| <6.5 | 0.89 (0.63, 1.27) | 0.528 |  | **1.38 (1.19, 1.61)** | **<0.001** |  | **0.66 (0.47, 0.91)** | **0.012** |  | **0.33 (0.20, 0.54)** | **<0.001** |  | 1.01 (0.89, 1.14) | 0.924 |
| 6.5–9.9 | 1.00 [Reference] |  |  | 1.00 [Reference] |  |  | 1.00 [Reference] |  |  | 1.00 [Reference] |  |  | 1.00 [Reference] |  |
| 10.0–13.4 | 1.12 (0.80, 1.58) | 0.500 |  | **1.34 (1.14, 1.58)** | **<0.001** |  | **1.39 (1.02, 1.88)** | **0.034** |  | **2.27 (1.56, 3.29)** | **<0.001** |  | **1.42 (1.25, 1.60)** | **<0.001** |
| 13.5–16.9 | 0.97 (0.67, 1.41) | 0.879 |  | **1.46 (1.23, 1.73)** | **<0.001** |  | **1.70 (1.24, 2.34)** | **0.001** |  | **2.25 (1.47, 3.44)** | **<0.001** |  | **1.50 (1.32, 1.72)** | **<0.001** |
| 17.0–19.9 | 1.00 (0.60, 1.67) | 0.998 |  | **1.94 (1.56, 2.40)** | **<0.001** |  | **3.01 (2.10, 4.30)** | **<0.001** |  | **3.66 (2.30, 5.82)** | **<0.001** |  | **2.16 (1.84, 2.55)** | **<0.001** |
| ≥ 20.0 | 0.86 (0.48, 1.53) | 0.606 |  | **4.31 (3.59, 5.17)** | **<0.001** |  | **8.96 (6.84, 11.75)** | **<0.001** |  | **7.34 (5.18, 10.41)** | **<0.001** |  | **5.17 (4.54, 5.88)** | **<0.001** |

Data are from China, 2015 to 2018. * Adjusted relative risks (95% CI) for preeclampsia were calculated with respect to categories of gestational weight gain stratified by prepregnancy BMI groups by performing robust Poisson regression models, which adjusted for basic characteristics of women, including year, region, age, education, ethnic origin, Hukou, assisted reproductive technology, and primigravida. † Additionally adjusted for prepregnancy BMI groups. BMI, body mass index; CI, confidence interval; RR, relative risk. Bold number refers to the value of RRs with 95% CIs statistically significant.
